# Human umbilical cord-derived mesenchymal stem cells protect against experimental colitis via CD5^+^ B regulatory cells

**DOI:** 10.1186/s13287-016-0376-2

**Published:** 2016-08-11

**Authors:** Kang Chao, Shenghong Zhang, Yun Qiu, Xiaoyong Chen, Xiaoran Zhang, Chuang Cai, Yanwen Peng, Ren Mao, Meirav Pevsner-Fischer, Shomron Ben-horin, Eran Elinav, Zhirong Zeng, Baili Chen, Yao He, Andy Peng Xiang, Minhu Chen

**Affiliations:** 1 Division of Gastroenterology, The First Affiliated Hospital, Sun Yat-sen University, Guangzhou, 510080 People’s Republic of China; 2 Division of Gastroenterology, The Sixth Affiliated Hospital, Sun Yat-sen University, Guangzhou, 510655 People’s Republic of China; 3 Center for Stem Cell Biology and Tissue Engineering, The Key Laboratory for Stem Cells and Tissue Engineering, Ministry of Education, Sun Yat-Sen University, Guangzhou, 510080 People’s Republic of China; 4 Department of Immunology, Weizmann Institute of Science, Rehovot, 7610001 Israel

**Keywords:** Mesenchymal stem cells, Colitis, Crohn’s disease, B regulatory cell, T helper cell

## Abstract

**Background:**

To clarify the effect of human umbilical cord-derived mesenchymal stem cell (hUC-MSCs) treatment on colitis and to explore the role of CD5^+^ B cells in MSC therapy.

**Methods:**

The trinitrobenzenesulfonic acid (TNBS)-induced colitis mouse model was used. HUC-MSCs were transferred peritoneally. Survival rates, colitis symptoms, and macroscopic and histologic scores were evaluated. CD4^+^ T helper (Th) cell subgroups and CD5^+^ regulatory B cell (Bregs) in lymphocytes were quantitated by flow cytometry. Cytokine levels were detected by ELISA and Bio-plex. CD5^+^ B cells were isolated for in vitro co-culture and adaptive transfer.

**Results:**

HUC-MSC treatment alleviated TNBS-induced colitis by increasing survival rates, relieving symptoms, and improving macroscopic and histologic scores. Labeled hUC-MSCs were located in the inflamed areas of colitis mice. Increases in regulatory T cells (Tregs) and CD5^+^ B cells and decreases in Th1 cells, Th17 cells, and several pro-inflammatory cytokines were observed with hUC-MSC treatment. After adaptive transfer, CD5^+^ B cells, which were located mainly in the peritoneal lavage fluid, improved TNBS-induced colitis by correcting Treg/Th1/Th17 imbalances. CD5^+^ B cells also inhibited T-cell proliferation and produced interleukin (IL)-10.

**Conclusions:**

HUC-MSCs protected against experimental colitis by boosting the numbers of CD5^+^ B cells and IL-10-producing CD5^+^ Bregs, and correcting Treg/Th17/Th1 imbalances.

**Electronic supplementary material:**

The online version of this article (doi:10.1186/s13287-016-0376-2) contains supplementary material, which is available to authorized users.

## Background

Crohn’s disease (CD) is a chronic, recurrent inflammatory disease of the gastrointestinal tract and is characterized by T-cell dysfunction, altered cytokine production, and cellular inflammation. These factors ultimately lead to mucosal damage of the alimentary tract. Although the etiology of CD remains unknown, there is substantial evidence showing that a failure of the mucosal immune system plays a key role in CD, especially the imbalance between effector T cells and suppressive regulatory T cells (Tregs). This imbalance results in the expansion of self-reactive T cells and inflammation [1]. Therefore, many available therapies and new drugs on the pipeline target inflammation-associated pathways. However, these therapies are not effective enough, as they are mostly nonspecific and can cause multiple adverse effects. This illustrates the need for novel therapeutic approaches and specific therapies that focus on immune regulation. The restoration of immune tolerance by the re-establishment of Treg/T helper (Th) cells imbalances has been proposed as an attractive therapeutic approach for CD. Stem cell therapy for CD has attracted attention since 1993, when the first case report of stem cell therapy in a CD patient was published [2]. Many case series and pilot clinical trials have demonstrated the efficacy of stem cell therapy, but with much uncertainty [3].

Mesenchymal stem cells (MSCs) are mesoderm-derived, fibroblast-like somatic cells that reside in the stroma of solid organs and function as precursors of nonhematopoietic connective tissues [4]. Recent studies have shown that MSCs are effective and safe in clinical trials of various pathologies, including graft-versus-host diseases (GVHD), rheumatic diseases, and inflammatory bowel diseases [5–8]. The mechanisms involved in these trials included the inhibition of T-cell proliferation, B-cell function, and dendritic cell maturation via the secretion of soluble factors by MSCs [9]. Apart from bone marrow-derived MSCs (BM-MSCs), which are the most widely used MSCs, other major sources of human MSCs are the umbilical cord, peripheral blood, and adipose tissue. Due to difficulties in obtaining sufficient autologous BM-MSCs, human MSCs obtained from the umbilical cord (hUC-MSCs) have recently emerged as an attractive alternative for cell therapy. In addition to its “immune-privileged” status and immunomodulatory properties, hUC-MSCs are easier to collect and expand in vitro [10, 11], thus making it a potentially promising tool in clinical applications.

Previous studies have focused on the effect of MSCs on T cells; however, recent studies found that a new regulatory subset, B regulatory cells (Bregs), could also play an important role. For example, a recent study focusing on an animal model of experimental autoimmune encephalomyelitis (EAE) found that the number of CD5^+^ Bregs increased after MSC therapy [12]. Our recent study involving BM-MSCs for GVHD patients also showed this phenomenon [13]. Therefore, we conducted this study to clarify the effect of hUC-MSCs on the treatment of experimental colitis in mice and to also explore the role of CD5^+^ B cells in hUC-MSC therapy.

## Methods

### Cell preparation

Human umbilical cords from full-term Caesarean section patients were collected upon delivery, stored in Dulbecco’s modified Eagle medium (DMEM)/F12 (1:1) culture medium, which was supplemented with 100 U/ml penicillin and 100 μg/ml streptomycin (GIBCO, Invitrogen Inc., Carlsbad, CA, USA), and transferred immediately for cell isolation, according to a previously described protocol [14]. Briefly, the cord was cut into pieces that were 4–5 cm long, and the vessels were pulled away to isolate Wharton’s Jelly (WJ). WJ was cut into 1–2-mm^3^ pieces and digested with 1 mg/ml collagenase II (Millipore Sigma, St. Louis, MO, USA) with phosphate-buffered saline (PBS) at 37 °C for 45 min. The digested mixture was then passed through a 100-μm filter (BD Biosciences, Franklin Lakes, NJ, USA) to obtain cell suspensions. The cells were washed with PBS solution and then cultured in DMEM/F12 medium containing 10 % fetal bovine serum, 2 mmol/L glutamine, 1 % nonessential amino acids, and 1 % penicillin/streptomycin (GIBCO, Invitrogen Inc., Carlsbad, CA, USA) at 37 °C and 5 % CO_2_. Nonadherent cells were removed by changing the medium after 3 days. Cells were expanded and identified according to the current statement of the International Society for Cellular Therapy (ISCT) [15]. Briefly, a minimal set of three standard criteria was used as the uniform definition of multipotent MSCs: adherence to plastic, specific surface antigen expression, and multipotent differentiation potential. The phenotype of multipotent MSCs is defined to be, at a minimum, the cell surface co-expression of antigens (CD105, CD73, and CD90 [≥95 % positive]) and the absence of hematopoietic lineage markers (CD45, CD34, CD14, CD19, and HLA-DR [≤2 % positive]). The surface marker was defined by the BD Stemflow hMSC Analysis Kit (BD Biosciences, Franklin Lakes, NJ, USA) containing pre-conjugated and pre-titrated cocktails of ISCT-defined positive expression markers (CD105 PerCP-Cy™5.5/CD73 APC/CD90 FITC) and negative expression markers (CD45/CD34/CD11b/CD19/HLA-DR PE). The multipotent differentiation potential of the isolated cells was identified using the Human Mesenchymal Stem Cell Functional Identification Kit (R&D, Minneapolis, MN, USA). Briefly, hUC-MSCs were seeded at 2 × 10^4^ cells/cm^2^ in StemXVivo Osteogenic/Adipogenic Base Media. And after 24 hours, the medium was replaced with adipogenic differentiation medium to induce adipogenesis. HUC-MSCs were seeded at 4.2 × 10^3^ cells/cm^2^ in StemXVivo Osteogenic/Adipogenic Base Media. When cells were to 50–70 % confluency, the medium was replaced by osteogenic differentiation medium. Differentiation medium was replaced every 3 days, and after 3 weeks cells were fixed in 10 % formalin and processed for histochemical analysis. Adipogenic differentiation was detected by oil red staining, and osteogenic differentiation was analyzed by alizarin red staining. This project was approved by the Human Ethics Committee of The First Affiliated Hospital at Sun Yat-sen University, and written informed consent was obtained for umbilical cord collections.

### Induction of colitis and cell transplantation

Colitis was induced in specific pathogen-free male BALB/c mice (6–8 weeks old), according to a previously described method [16]. All experiments were performed according to the Institutional Guidelines for the Care and Use of Laboratory Animals in Research and were approved by the Ethics Committee at Sun Yat-sen University. Briefly, mice were pre-sensitized with the trinitrobenzenesulfonic acid (TNBS) pre-sensitization solution on day 1. The pre-sensitization solution was prepared by mixing acetone and olive oil in a 4:1 volume ratio by rigorous vortexing and then mixing 4 volumes of acetone/olive oil with 1 volume of 5 % TNBS solution to obtain 1 % (w/v) TNBS. Control mice were treated with the pre-sensitization solution without TNBS. BALB/c mice were lightly anesthetized after a 24-hour fast on day 8. To induce colitis, 5 % TNBS (Millipore Sigma, St. Louis, MO) in 50 % ethanol (2.5 mg/kg TNBS) was administered intrarectally via a 3.5 French (F) catheter equipped with a 1-ml syringe. The catheter was inserted into the rectum until the tip was advanced to 4 cm proximal to the anal verge. Control mice received 50 % ethanol alone. Passages 3–5 of hUC-MSCs were used for cell transplantation. BALB/c mice were treated intraperitoneally either with the medium as the control or with 10^6^ hUC-MSCs/mouse 2 hours after instillation of TNBS.

### Assessment of colitis severity

Animals were monitored for the appearance of diarrhea, body weight loss, and survival every day for a total of 14 days. The baseline data were collected before instillation of TNBS. Disease activity and histologic scores were evaluated as previously described [17]. For disease activity, a score system containing percentage of weight loss, stool consistency, and fecal occult blood test was used [16, 17]. For histopathology analysis, a colon specimen from the middle part (1 cm to the anus to cecum) was fixed in 10 % buffered formalin phosphate and then embedded in paraffin. Sections were stained with hematoxylin and eosin, and inflammation was graded from 0–4 as follows, in a blinded fashion: 0, no signs of inflammation; 1, low leukocyte infiltration; 2, moderate leukocyte infiltration; 3, high leukocyte infiltration, moderate fibrosis, high vascular density, thickening of the colon wall, moderate goblet cell loss and focal loss of crypts; and 4, transmural infiltrations, massive loss of goblet cells, extensive fibrosis, and diffuse loss of crypts. Myeloperoxidase (MPO) activity was assessed by the MPO kit (Jiancheng, Nanjing, China), according to the manufacturer’s instructions. For survival and colitis score analysis, there were 20 mice in the model and treatment groups and 10 mice for the control and naïve groups. For histological and immunological analysis, mice were sacrificed at day 3 after colitis induction, at the peak of inflammation (*n* = 9 for each group).

### In vivo imaging

MSCs were traced in vivo with the Renilla Luciferase Assay System (Promega, Madison, WI, USA). First, the EGFP-luciferase system was conducted and transferred to MSCs. Cell transplantation was conducted as described above. Renilla luciferase substrate was intraperitoneally injected after cell transfer at different time points (day 1, day 3, and day 5). Using the Xenogen IVIS Spectrum in vivo visible light system (Caliper Life Sciences, Hopkinton, MA, USA), cell tracing was performed at about 10 min after substrate injection.

### Immunologic analysis of T- and B-cell subsets in the mesenteric lymph node (MLN) and spleen

Colitis mice were sacrificed at day 3 after colitis induction, at the peak of inflammation. Lymphocytes of MLN cells and the spleen were isolated through a 100-μm filter (BD Biosciences, Franklin Lakes, NJ, USA). Lymphocytes were then suspended at a density of 2 × 10^6^ cells/ml in RPMI 1640 culture medium, which was supplemented with 100 U/ml penicillin, 100 μg/ml streptomycin, 2 mmol/L glutamine, and 10 % heat-inactivated fetal calf serum (GIBCO, Invitrogen Inc., Carlsbad, CA, USA). To identify Tregs, 2 × 10^6^ lymphocytes were surface-labeled with phycoerythrin (PE)-labeled anti-CD4 and allophycocyanin (APC)-cyanine (Cy)7-labeled Foxp3. For Th1/Th2/Th17 cell subgroup analyses, 2 × 10^6^ cells were stimulated with 50 ng/ml phorbol myristate acetate and 1 mmol/L ionomycin (Millipore Sigma, St. Louis, MO, USA) for 4 hours in the presence of monensin (BD Biosciences, Franklin Lakes, NJ, USA). The incubator was set at 37 °C under a 5 % CO_2_ atmosphere. After 4 hours, intracellular staining was performed with APC-labeled anti-CD4, PE-labeled anti-interleukin (IL)-4, PE-labeled anti-IL-17, and FITC-labeled anti-interferon (IFN)-γ (BD Biosciences, Franklin Lakes, NJ, USA). For CD5 cell subgroup analysis, cells were surface-labeled with FITC-labeled CD5 and PE-CY7-labeled CD19. Flow cytometry was carried out using a BD FACScan (BD Biosciences, Franklin Lakes, NJ, USA), in which 300,000–500,000 events were collected, and lymphocytes were gated based on their forward and side light scatter properties. Data were analyzed using the Gallios Flow Cytometer (Beckman Coulter, Brea, CA, USA) and Kaluza Analysis software. The proportion of Tregs was determined based on CD4^+^Foxp3. CD4^+^IFN-γ^+^, CD4^+^IL-4^+^, CD4^+^IL-17^+^, and CD5 + CD19+ cells were defined as Th1 cells, Th2 cells, Th17 cells, and CD5^+^ Bregs, respectively. For cytokine expression, the serum was separated from peripheral blood collected through tail vein on day 3. Tumor necrosis factor (TNF)-α, IL-12, IL-6, IL-23, IL-21, IFN-γ, and IL-17A were detected by the ProcartaPlex™ Multiplex Immunoassays kit (eBioscience, Santa Clara, CA, USA) and Bio-plex system (Bio-Rad, Hercules, CA, USA). Transforming growth factor (TGF)-β was detected by measured by an enzyme-linked immunosorbent assay (ELISA) (eBioscience, Santa Clara, CA, USA), according to manufacturer instructions. All assays were performed in triplicate wells per condition in each experiment.

### In vitro and in vivo study of CD5^+^ Bregs

CD5^+^ B cells have been shown to exist in the peritoneal cavity [18]. Thus, CD5^+^ B cells were also analyzed in peritoneal lavage fluid to clarify its distribution in hUC-MSC-treated colitis mice. CD5^+^ B cells were isolated by flow cytometry and co-cultured with carboxyfluorescein succinimidyl ester (CFSE) (Invitrogen, Inc., Carlsbad, CA, USA)-labeled T cells. Cell proliferation was then detected by flow cytometry (Beckman Coulter, Brea, CA, USA). For the in vivo functional study, CD5^+^ B cells were isolated form spleen lymphocytes by flow cytometry. Isolated CD5^+^ B cells were transplanted through the tail vein of TNBS-induced colitis mice on day 3, in the peak of the inflammation. Disease severity and T-cell subgroups were analyzed according to the methods described above.

## Results

### Identification of hUC-MSCs

The hUC-MSCs showed a fibroblast-like morphology, expressed certain antigens (CD105, CD73, and CD90 [≥95 % positive]), and lacked hematopoietic lineage markers (CD45, CD34, CD14, CD19, and HLA-DR [≤2 % positive]). After certain inducing environments of osteogenesis and adiposis, the cells had the ability to differentiate multidirectionally to osteogenesis and adiposis (Additional file 1: Figure S1).

### HUC-MSC therapy protected against TNBS-induced colitis

**Fig. 1:**
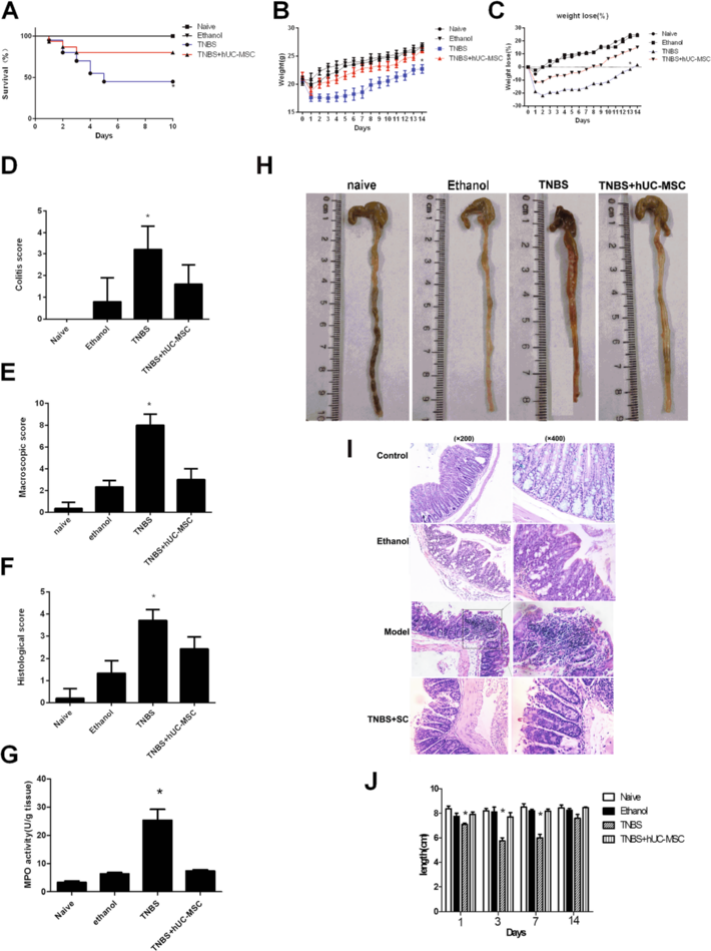
Human umbilical cord-derived mesenchymal stem cell (hUC-MSC) therapy protects against TNBS-induced colitis. HUC-MSC therapy increased the survival rate of experimental colitis mice (**a**), decreased weight loss (**b** and **c**), alleviated colitis symptoms (**d**), and improved macroscopic (**e**) and histologic (**f**) scores. Myeloperoxidase (MPO) activity is shown in (**g**), and pictures of the colon (**h**) with hematoxylin and eosin staining (**i**) of each group are presented. The colon length which can reflect the inflammation was shown in (**j**). *n* = 20 for colitis model and treatment groups; *n* = 10 for model control and naïve mice; ^*^ *P* < 0.05 vs. MSC-treated mice. *TNBS* trinitrobenzenesulfonic acid

TNBS-treated mice developed a severe illness, which was characterized by bloody diarrhea, rectal prolapse, pancolitis, and sustained weight loss. The mortality of the colitis model was 55 %, whereas the rates were 20 % in hUC-MSC-treated TNBS mice and 0 % in ethanol and naïve controls (Fig. 1a). Similar to ethanol control mice, hUC-MSC-treated mice had rapidly recovered body weight loss (b-c) and milder inflammation. They also exhibited significantly lower colitis, decreased macroscopic and histologic scores (Fig. 1d–f), and less neutrophil infiltration, as reflected by lower MPO activity (Fig. 1g). Macroscopic examination of TNBS colons showed hyperemia, edema, and inflammation that were significantly more severe than that in HUC-MSC-treated mice (Fig. 1h, j). Histologic examination of the colons showed that HUC-MSC treatment reduced the TNBS-induced inflammation of the transmural area, depletion of epithelial cells, and focal loss of crypts (Fig. 1i).

### HUC-MSCs may migrate to the inflamed areas

**Fig. 2:**
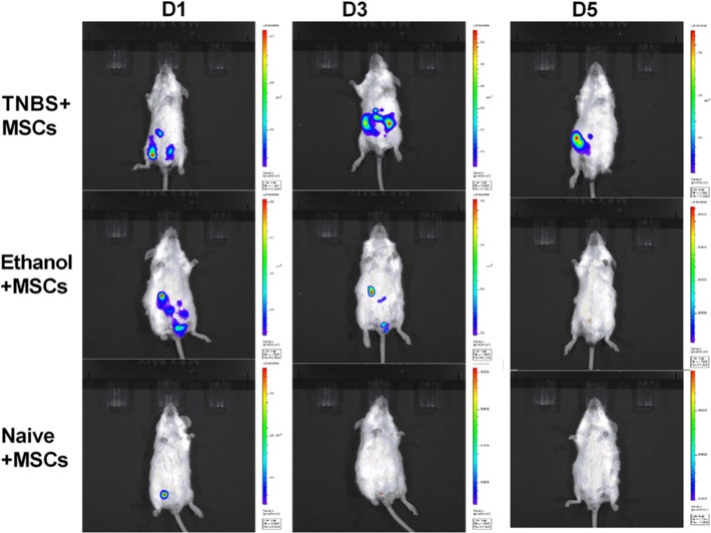
MSCs migrate to the inflamed areas. In vivo tracing of MSCs on days 1, 3, and 5, the labeled cells were detected by the imaging system. *Warmer colors* indicate more accumulation of cells. *MSCs* mesenchymal stem cells, *TNBS* trinitrobenzenesulfonic acid

By in vivo cell tracing, we found that hUC-MSCs accumulated in the peritoneal cavity of TNBS and ethanol mice on day 1 (6 hours after colitis induction), whereas only a few cells that were limited to the site of cell injection could be found in naïve mice. At the peak of colitis, the cells still accumulated in the abdomen of TNBS mice but could not be detected in ethanol and naïve mice, thus suggesting relevance with colon inflammation. At day 5, when recovery from colitis began, the number of hUC-MSCs gradually decreased and could not be traced (Fig. 2). This phenomenon indicated that the MSCs may migrate to the inflamed area and be associated with the degree of inflammation.

### HUC-MSCs altered Th cell and Treg imbalance in colitis mice

**Fig. 3:**
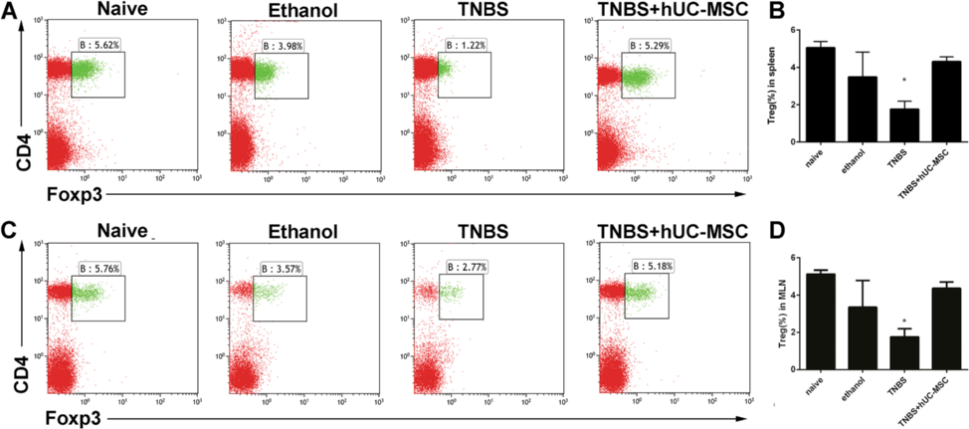
hUC-MSCs alter numbers of regulatory T cells (Tregs) in colitis mice. Lymphocytes were co-stained with anti-CD4 and anti-FoxP3 antibodies and evaluated by flow cytometry. Tregs were defined as CD4^+^FoxP3^+^. The frequency of Tregs from the hUC-MSC-treated group was significantly lower than that in controls. Representative dot plots of Tregs in the spleen (**a**) and mesenteric lymph node (MLN) (**c**) of each group. Treg proportions are shown in (**b**) and (**d**). Data are presented as plots with *P* value. *n* = 9 for each group; ^*^ *P* < 0.05 vs. MSC-treated mice. *hUC-MSC* human umbilical cord-derived mesenchymal stem cell, *TNBS* trinitrobenzenesulfonic acid

**Fig. 4:**
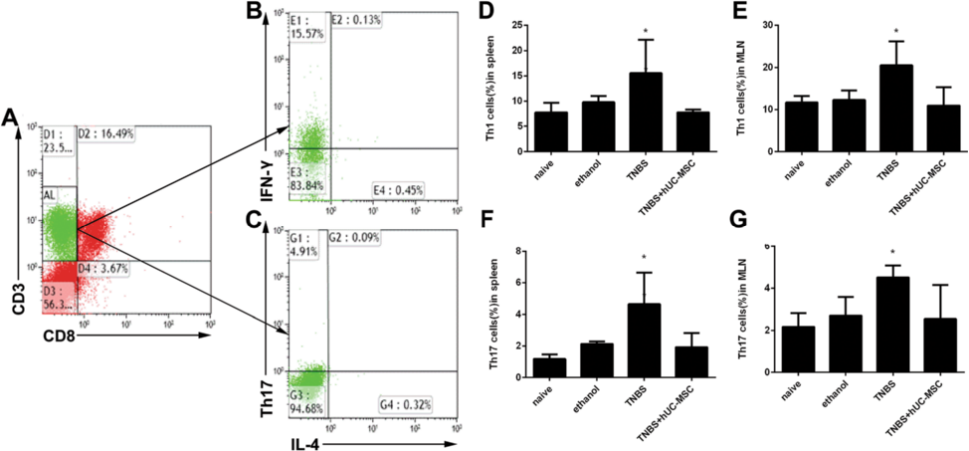
hUC-MSCs alter T helper cell subgroups in colitis mice. Populations of Th1/Th2/Th17 cells as a proportion of total CD4^+^ cells were evaluated by flow cytometry. Cells were co-stained with antibodies against CD3, CD8, interferon (IFN)-γ, interleukin (IL)-4, or IL-17 (CD4^+^ cells). CD3^+^CD8^-^ cells were gated (**a**). CD4^+^IFN-γ^+^, CD4^+^IL-4^+^, and CD4^+^IL-17^+^ cells were defined as Th1, Th2, and Th17 cells, respectively. Representative dot plots are shown in panels **b**–**c**. Proportions of Th1 and Th17 cells in the four participant groups are shown in panels **d**–**g**. Data are presented as plots with *P* value. *n* = 9 for each group; ^*^ *P* < 0.05 vs. MSC-treated mice. *hUC-MSC* human umbilical cord-derived mesenchymal stem cell, *TNBS* trinitrobenzenesulfonic acid

**Fig. 5:**
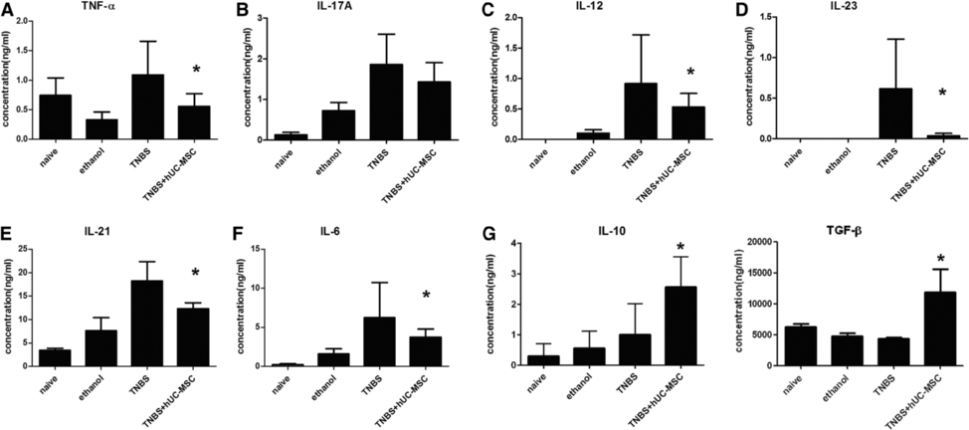
Serum cytokine expression in each group. Th1 cell-related cytokines (tumor necrosis factor [TNF]-α and interleukin [IL]-12) and Th17 cell-related cytokines (IL-6, IL-23, and IL-21) were decreased after cell transplantation. IL-10 and transforming growth factor (TGF)-β were increased after cell transplantation. For IL-17A, there was a decreased tendency (*P* = 0.09). *n* = 6 for each group; ^*^ *P* < 0.05 vs. TNBS-treated mice. *hUC-MSC* human umbilical cord-derived mesenchymal stem cell, *TNBS* trinitrobenzenesulfonic acid

We further used flow cytometry to analyze immunologic changes after hUC-MSC transplantation. In splenic lymphocytes, the Treg proportions were 4.31 ± 0.21 %, 1.77 ± 0.32 %, 3.49 ± 1.20 %, and 5.05 ± 0.23 % in hUC-MSC-treated mice, TNBS mice, ethanol control mice, and naïve mice, respectively. Similar tendencies in MLN lymphocytes were observed among groups (Fig. 3). Furthermore, there was a significant decrease in Th1 and Th17 cells in both splenic and MLN lymphocytes after hUC-MSC therapy (Fig. 4). Th2 cells were rarely expressed, and no differences were observed after cell transfer. Levels of pro-inflammatory cytokines, such as TNF-α, IL-12, IL-6, IL-23, and IL-21, decreased significantly in the plasma after MSC treatment (*P* < 0.05). IL-17A, which is the main cytokine of Th17 cells, showed a decreased tendency (*P* = 0.09) (Fig. 5). IL-10 and TGF-β, which are associated with immunosuppression, were significantly higher in hUC-MSC-treated mice (*P* = 0.04 and 0.02, respectively).

### CD5^+^ B cells alleviated colitis in mice in vivo by regulating T-cell responses

**Fig. 6:**
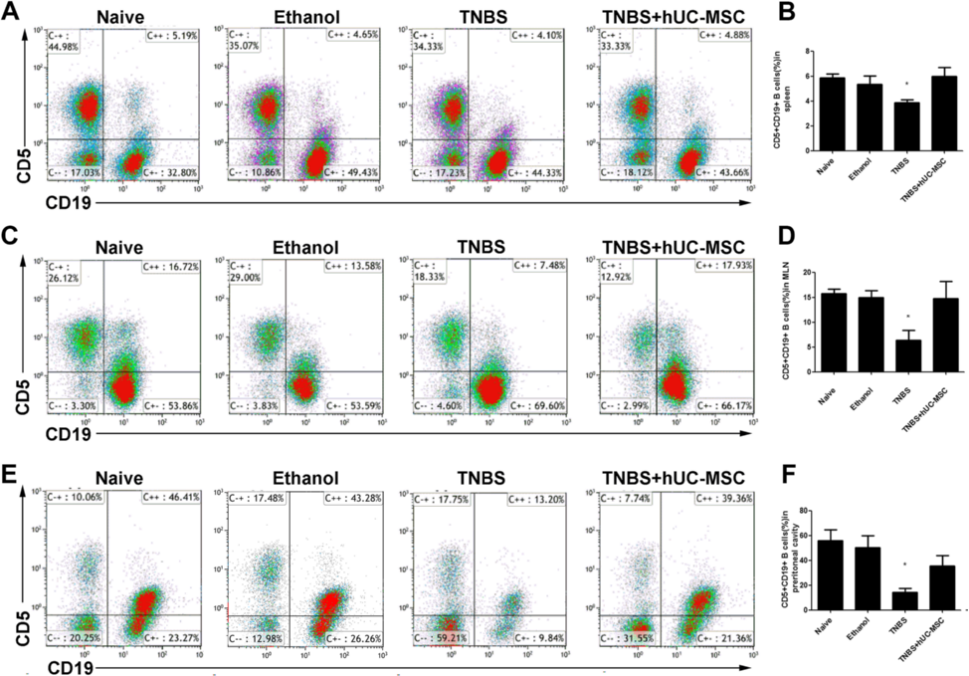
CD5^+^ B cells significantly increase after hUC-MSC therapy. Populations of CD5^+^ B cells were identified as CD5^+^CD19^+^ by flow cytometry. Representative dot plots of CD5^+^ B cells in the spleen (**a**), mesenteric lymph node (MLN) (**c**), and peritoneal cavity (**e**) are shown. Data are presented as plots with *P* value (**b**, **d** and **f**). *n* = 9 for each group; ^*^ *P* < 0.05 vs. MSC-treated mice. *hUC-MSC* human umbilical cord-derived mesenchymal stem cell, *TNBS* trinitrobenzenesulfonic acid

We found a significant increase in CD5^+^ B cells after cell transplantation in both splenic and MLN lymphocytes (Fig. 6), suggesting that CD5^+^ B cells might play a role in immune regulation. Interestingly, CD5^+^ B cells mainly distributed in the peritoneal cavity and decreased significantly in the colitis model; this was reversed by hUC-MSC therapy (Fig. 6). The above phenomenon led us to hypothesize that CD5^+^ B cells could regulate T-cell imbalance.

**Fig. 7:**
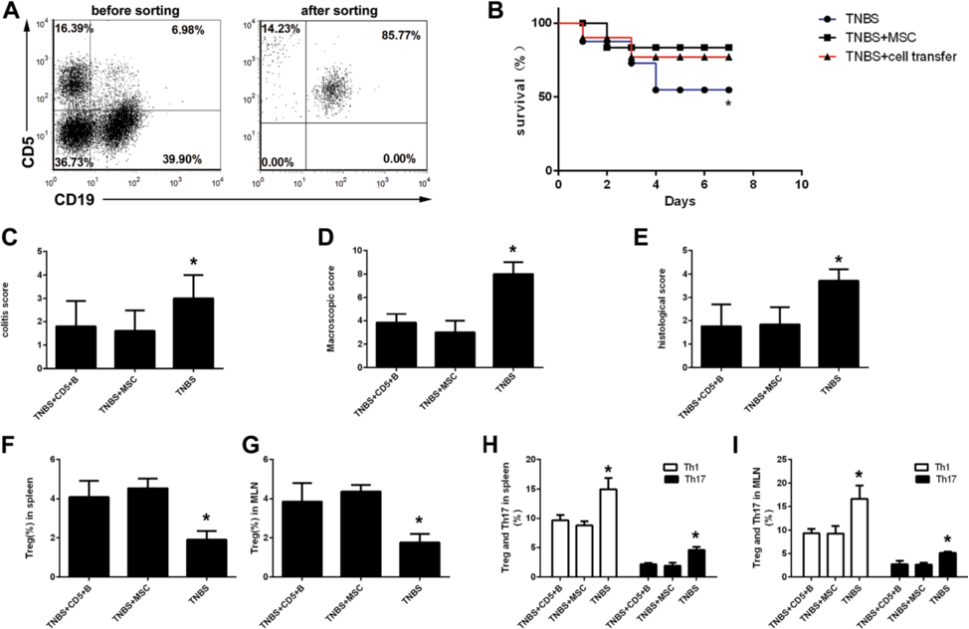
Adaptive transfer of CD5^+^ B cells alleviates TNBS-induced colitis. Sorted CD5^+^ B cells (**a**) were used for transplantation. After adaptive transfer, the cells showed similar efficiency in colitis mice as hUC-MSCs (**b**–**e**), and this effect was associated with Treg/Th imbalances (**f**–**i**). *hUC-MSC* human umbilical cord-derived mesenchymal stem cell, *MLN* mesenteric lymph node, *Th* T helper, *TNBS* trinitrobenzenesulfonic acid

**Fig. 8:**
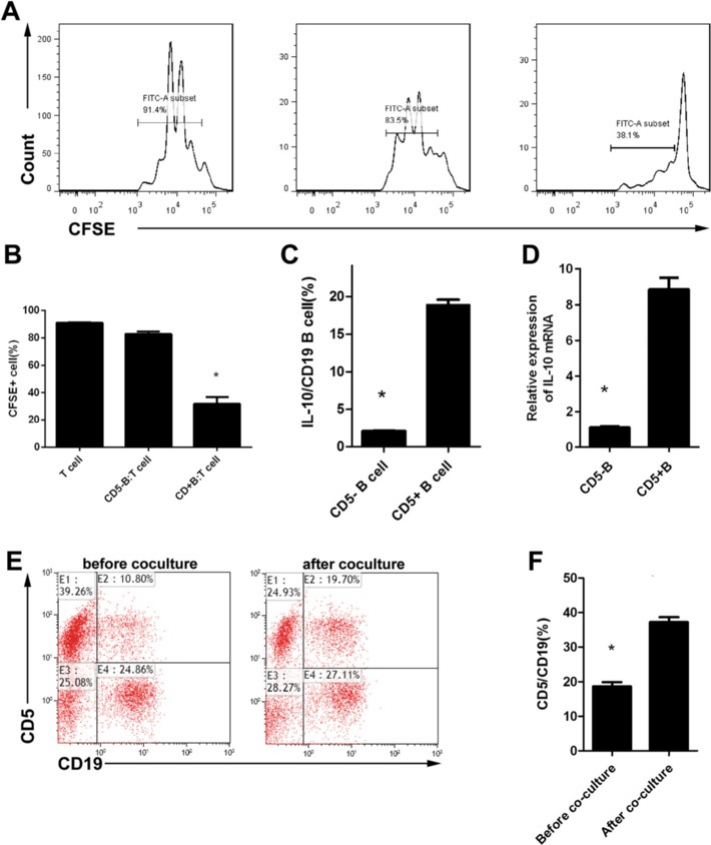
CD5^+^ B cells inhibit T-cell differentiation and are induced by hUC-MSCs. **a**–**b** CD5^+^ B cells inhibited carboxyfluorescein succinimidyl ester (CFSE)-labeled T cells. **c**–**d** CD5 + B cells express high level of interleukin (IL)-10, while CD5-B cells express significant lower level of IL-10 both in cell frequency and mRNA level. **e**–**f** CD5^+^ B cells were induced in vitro after co-culture with hUC-MSCs

To further clarify the function of CD5^+^ B cells, we conducted both in vivo and in vitro studies. Adaptive transfer of isolated CD5^+^ B cells had the same effect as hUC-MSC therapy (Fig. 7) and resulted in increased survival, decreased disease activity, and lower macroscopic and histologic scores. Interestingly, this effect was associated with an alteration of Th/Treg balances (Fig. 7). The in vitro co-culture of hUC-MSCs and splenic lymphocytes significantly increased the number of CD5^+^ B cells (Fig. 8). When co-cultured with CFSE-labeled T cells, CD5^+^ B cells could inhibit T-cell proliferation and may be associated with IL-10 (Fig. 8).

## Discussion

In the past decades, new drugs for CD have greatly improved the efficacy and quality of life of CD patients. However, even in the era of biologics, the fight against CD is far from over, as there is still no ideal treatment or cure. Findings from recent studies have confirmed that immunologic factors play a central role in the pathogenesis of CD. We have previously found imbalances among CD4^+^ T-cell subgroups in Chinese CD patients. Treg/Th1 and Treg/Th17 ratios are associated with disease activity and are potential prognostic indicators for predicting CD recurrence [19]. Thus, new treatments targeting immune imbalances over specific cytokines appear to be the best therapeutic candidates for CD.

MSC therapy has been promising in several inflammatory diseases, including CD. Th1 and Treg imbalances play a central role [8, 17, 20–24] in CD. However, up to now, there has only been one report concerning the effect of hUC-MSCs on experimental colitis. This report showed that hUC-MSCSs could modulate Treg/Th1/Th17 imbalances [14]. Similarly, we showed that hUC-MSCs indeed alleviated experimental colitis. We also confirmed an alteration of Treg/Th1/Th17 imbalances and cytokine production after cell transplantation. Strikingly, the number of CD5^+^ B cells increased significantly after hUC-MSC therapy. In previous studies, Bregs showed an immunosuppressive role via the expression of CD5 and secretion of IL-10 [25–29]. Although there are controversies concerning the definition and surface marker of Bregs, this subset is known to exert immunosuppressive functions by regulating Th cells and Tregs, inducing T-cell and B-cell apoptosis, and inhibiting other immune-related cells, including CD8^+^ T cells and natural killer T cells. Furthermore, Bregs are associated with autoimmune diseases, such as type I diabetes, GVHD, arthritis, and lupus [29, 30]. In colitis models, Bregs can exert anti-inflammatory effects [27, 28, 31]. In a T-cell receptor-α^-/-^ colitis model, B-cell depletion resulted in severe colitis, whereas the adaptive transfer of B cells alleviated the colitis symptoms. When CD1d was simultaneously knocked out, the symptoms of colitis became more severe, thereby suggesting that the B-cell subset can regulate inflammation in the colitis models [27]. In IL-10^-/-^ experimental colitis models, IL-10-expressing B cells alleviate colitis [28]. Only two studies have reported increases in the numbers of CD5^+^ B cells after BM-MSC transplantation. One study involved the EAE model and revealed an increase in numbers of CD5^+^ B cells after BM-MSC transplantation [12], however, this study did not further analyze the potential mechanisms and function of CD5 + B cells. The other study from our team revealed that the CD5^+^ B subset is associated with the efficiency of BM-MSC therapy in GVHD [13]. In the present study, in vivo cell tracing results show that the cells exerted their function specifically in the inflamed areas. As CD5^+^ B cells mainly reside in the peritoneum, we analyzed the proportion of CD5^+^ B cells in peritoneal lavage fluid, splenic lymphocytes, and MLN lymphocytes. Numbers of CD5^+^ B cells were significantly elevated after hUC-MSC transfer. We also conducted further in vivo and in vitro studies to verify whether MSCs can regulate CD5^+^ B cells to modulate the immune status of the colitis model. Upon co-culture with hUC-MSCs, CD5^+^ B cells increased and inhibited T-cell proliferation in vitro. In addition, the adaptive transfer of CD5^+^ B cells into colitis mice alleviated colitis in a similar manner as that achieved with hUC-MSCs. Importantly, this effect was associated with the alteration of Th/Treg imbalances. CD5^+^ Bregs also inhibited T-cell proliferation in vitro. These findings suggest that CD5^+^ B cells can exert a regulatory effect and protect against TNBS-induced colitis by regulating T-cell balances. Therefore, the effect of CD5^+^ B cells may be a new mechanism of MSC therapy in CD. Furthermore, CD5^+^ B cells produced more IL-10 mRNA and protein, thus indicating that IL-10 may be an important factor that coordinates the immunoregulatory effect of MSC-induced Bregs. It should be emphasized that CD5 is also highly expressed on B-cell chronic lymphocytic leukemia (B-CLL). The specific surface marker ROR1 [32] and receptor of lysophosphatidic acid (LPA) [33, 34] are the markers of B-CLL but not normal B cell. To differentiate the immunosuppressive B cells form B-CLL, we isolated the CD5^+^B cell from naïve, model and MSC-treated mice and detected the expressions of LPA receptors (LPARs) by real-time PCR. We found a very low expression of LPARs (LPAR1-5) genes. Furthermore, the expressions of LPARs in naïve, model and MSC-treated mice were similar. Therefore, we concluded that the increased CD5 + B cell after MSCs transplantation were normal B cells.

We did in vivo cell tracking using the luciferase reporting system. We found that direct cell repair may be less important than immune regulation, as the cells could not be detected 5 days after cell transplantation. Interestingly, the cells mainly distributed in the abdomen when transferred peritoneally in the colitis model, while they could not be tracked in naïve mice. One possible reason is that the cells distributed to a wide range of tissues and organs in a random fashion in naïve mice, as shown in previous studies [14, 35]. But the IVIS system can track the cells when the signal is large enough where cells were aggregated together. However, we knew very little about the destination of the cells in our analysis. Therefore, a more specific three-dimensional option with high resolution was needed to further localize the cells in vivo.

This study has some limitations. First, we used the surface marker CD5^+^ to define Bregs and did not further clarify other Breg subsets with different surface markers. Second, we did not explain the mechanisms of how hUC-MSCs can induce CD5^+^ Breg differentiation. Further analysis is needed to clarify these questions.

## Conclusions

We show for the first time that hUC-MSCs could protect against experimental colitis in mice by correcting Treg/Th17/Th1 imbalances. The underlying mechanism possibly acts via boosting the numbers of CD5^+^ B cells and IL-10-producing CD5^+^ Bregs. Further analysis is needed to clarify the issue that hUC-MSCs can induce CD5^+^ Breg differentiation.

## Abbreviations

BM-MSCs, bone marrow-derived mesenchymal stem cells; Bregs, regulatory B cell; CD, Crohn’s disease; EAE, experimental autoimmune encephalomyelitis; GVHD, graft-versus-host diseases; hUC-MSCs, human umbilical cord-derived mesenchymal stem cells; IFN, interferon; IL, interleukin; MLN, mesenteric lymph node; MPO, myeloperoxidase; Th cell, T helper cell; TGF, transforming growth factor; TNF, tumor necrosis factor; Tregs, regulatory T cells; WJ, Wharton’s Jelly

## Additional file

Additional file 1: Figure S1. Identification of human umbilical cord-derived mesenchymal stem cells (hUC-MSCs). HUC-MSCs were identified according to the International Society for Cellular Therapy statement. The cells were adherent to plastic (A), differentiated to adipocytes (B) and osteoblasts (C) in vitro, and expressed specific surface antigens (positive for CD73, CD90, and CD105; negative for CD45, CD34, CD14, CD19, and HLA-DR) (D). (TIF 6040 kb)
