# Correction to: Human umbilical cord-derived mesenchymal stem cells protect against experimental colitis via CD5+ B regulatory cells

**DOI:** 10.1186/s13287-019-1132-1

**Published:** 2019-01-21

**Authors:** Kang Chao, Shenghong Zhang, Yun Qiu, Xiaoyong Chen, Xiaoran Zhang, Chuang Cai, Yanwen Peng, Ren Mao, Meirav Pevsner-Fischer, Shomron Ben-horin, Eran Elinav, Zhirong Zeng, Baili Chen, Yao He, Andy Peng Xiang, Minhu Chen

**Affiliations:** 1 Division of Gastroenterology, The First Affiliated Hospital, Sun Yat-sen University, Guangzhou, 510080 People’s Republic of China; 2 Division of Gastroenterology, The Sixth Affiliated Hospital, Sun Yat-sen University, Guangzhou, 510655 People’s Republic of China; 3 Center for Stem Cell Biology and Tissue Engineering, The Key Laboratory for Stem Cells and Tissue Engineering, Ministry of Education, Sun Yat-Sen University, Guangzhou, 510080 People’s Republic of China; 4 Department of Immunology, Weizmann Institute of Science, 7610001 Rehovot, Israel

**Correction to: Stem Cell Res Ther**

**https://doi.org/10.1186/s13287-016-0376-2**

The original article [1] contains a duplication error within Fig. 3. The correct version of Fig. 3 can instead be viewed directly ahead.

**Fig. 3:**
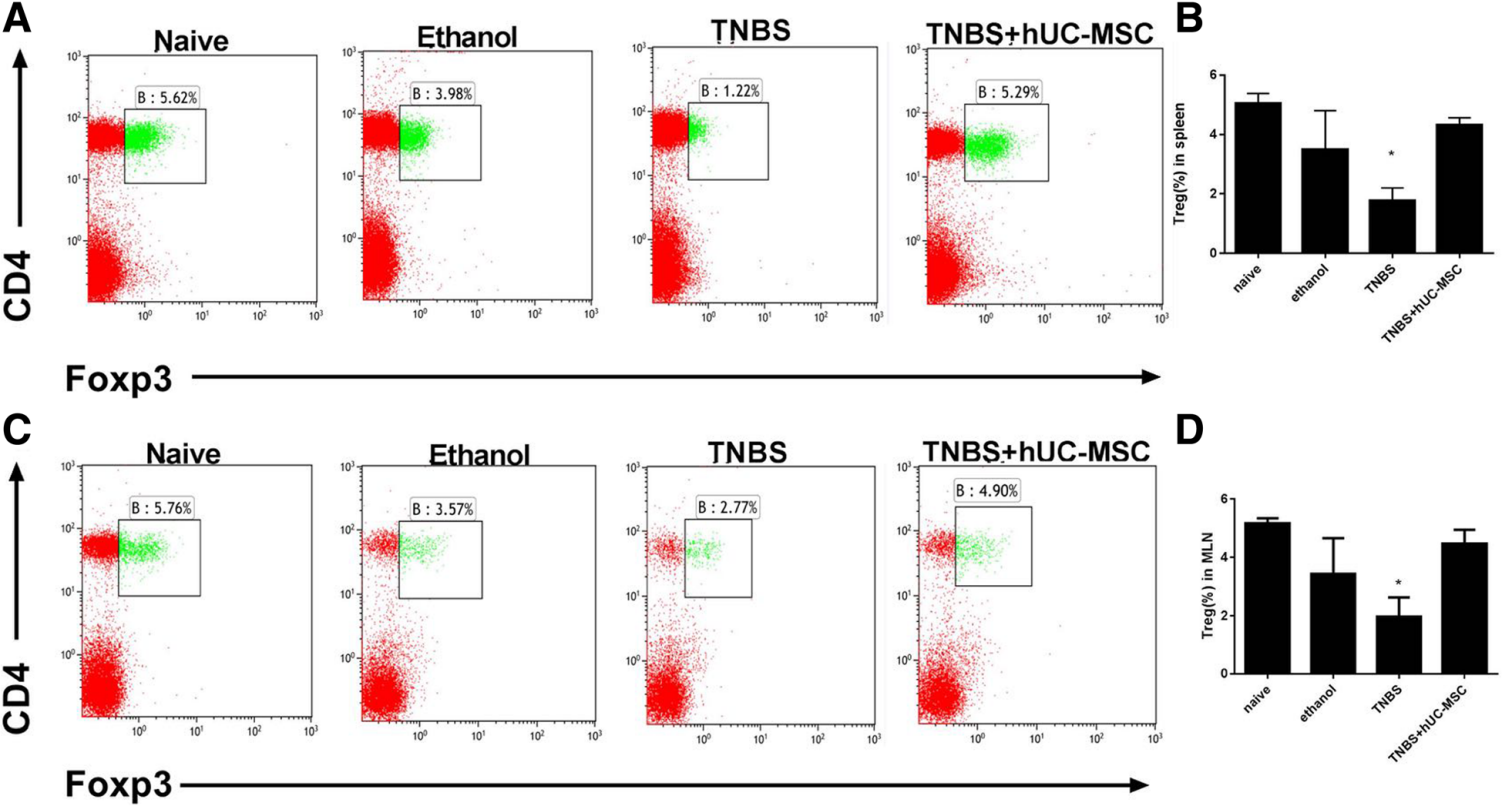
hUC-MSCs alter numbers of regulatory T cells (Tregs) in colitis mice. Lymphocytes were co-stained with anti-CD4 and anti-FoxP3 antibodies and evaluated by flow cytometry. Tregs were defined as CD4^+^FoxP3^+^. The frequency of Tregs from the hUC-MSC-treated group was significantly lower than that in controls. Representative dot plots of Tregs in the spleen (**a**) and mesenteric lymph node (MLN) (**c**) of each group. Treg proportions are shown in (**b**) and (**d**). Data are presented as plots with *P* value. *n* = 9 for each group; ^*^ *P* < 0.05 vs. MSC-treated mice. *hUC-MSC* human umbilical cord-derived mesenchymal stem cell, *TNBS* trinitrobenzenesulfonic acid
