# Supplementary figures and images for: Human umbilical cord-derived mesenchymal stem cells protect against experimental colitis via CD5+ B regulatory cells
Source: Stem Cell Res Ther. 2016 Aug 11;7:109. doi: 10.1186/s13287-016-0376-2 (PMC4981968; doi:10.1186/s13287-016-0376-2)

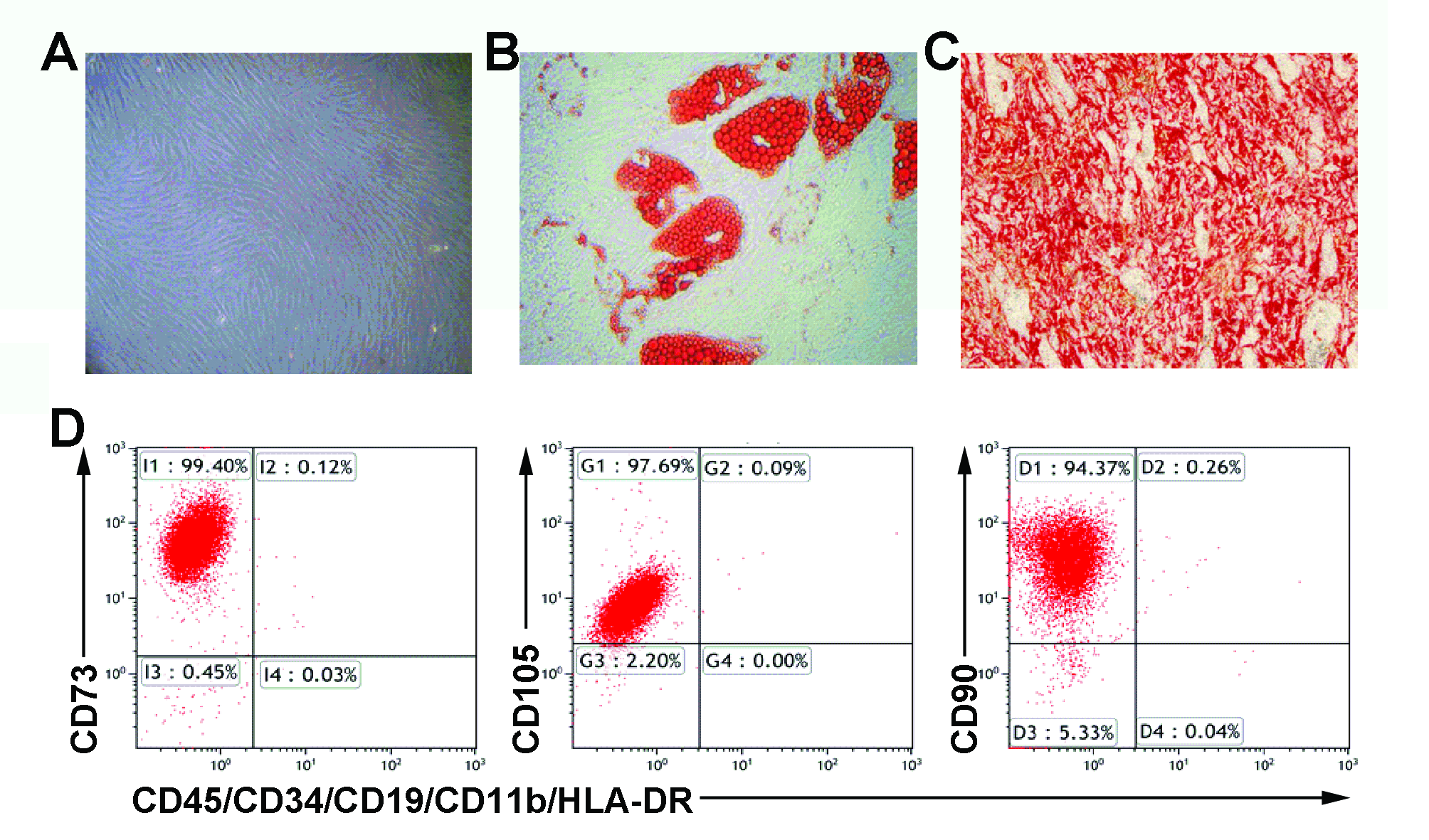

Supplement: Additional file 1: Figure S1. — Identification of human umbilical cord-derived mesenchymal stem cells (hUC-MSCs). HUC-MSCs were identified according to the International Society for Cellular Therapy statement. The cells were adherent to plastic (A), differentiated to adipocytes (B) and osteoblasts (C) in vitro, and expressed specific surface antigens (positive for CD73, CD90, and CD105; negative for CD45, CD34, CD14, CD19, and HLA-DR) (D). (TIF 6040 kb) [file 13287_2016_376_MOESM1_ESM.tif]
